# Surgical treatment of symptomatic neuromas: a feasibility study using the NEUROCAP® bioresorbable nerve capping device

**DOI:** 10.1177/17531934211022164

**Published:** 2021-06-09

**Authors:** Edwin L. de Vrij, Tim R. Schäfer, Tom van Mulken, Mariëtta J. O. E. Bertleff

**Affiliations:** 1 Department of Plastic Surgery, University Medical Center Groningen, Groningen, The Netherlands; 2 Department of Plastic Surgery, Maastricht University Medical Center, Maastricht, The Netherlands; 3 Department of Plastic Surgery, HandsOnCare Hand and Wrist Center Medical Center Group, Bosch en Duin, The Netherlands

Dear Editor,

Neuropathic symptoms secondary to neuromas, following traumatic or iatrogenic causes, can significantly affect the quality of life and socioeconomic integration of patients (van der Avoort et al., 2013). Many methods have been described to treat symptomatic end-neuromas, including pharmacological and surgical techniques, each with its own limitations. In general, surgical treatment involves excision of the neuroma and burying the nerve end within soft tissues or bone, with varying success rates. Repeat surgeries are often required (van der Avoort et al., 2013) and there is usually some degree of residual pain.

**Figure 1.**
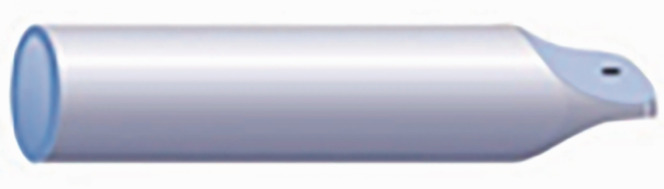
The NEUROCAP® device.

The use of synthetic nerve capping materials to limit pathophysiological axon regeneration have recently been developed to reduce the need for repeat surgeries. It is hypothesized that neural capping allows for epineural recovery within the device but reduces the regeneration of disorganized axons and formation of a bulbous mass that is susceptible to mechanical stimulation and painful adhesion to surrounding scar tissues (Yan et al., 2014). A recent study demonstrated NEUROCAP® (Polyganics, Groningen, Netherlands) to be effective in a case of digital end-neuroma (George et al., 2020). NEUROCAP® is a bioresorbable nerve cap (Figure 1), made of biocompatible co-polyester (Poly(68/32[15/85 D/L] Lactide-ε-Caprolactone)) or PLCL. In this cohort study, we assessed the safety, outcome and ease of use of NEUROCAP® to reduce the recurrence of peripheral symptomatic end-neuroma with a 12-month follow-up.

**Figure 2.**
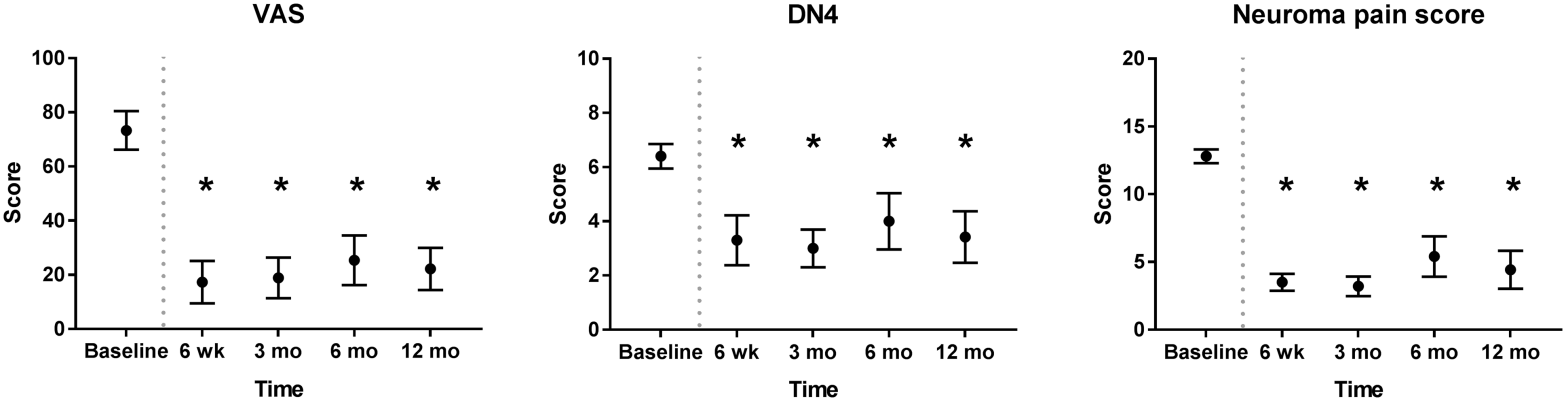
Pain reduction over time after neuroma excision and capping with NEUROCAP® as determined by visual analogue scale (VAS), Douleur Neuropathique 4 (DN4) questionnaire and neuroma pain score. Dotted grey line represents the time of treatment. Measurements at all time points were for 10 patients, except for 6 and 12 months (*n* = 9). Data represented as estimated marginal means with standard deviation. *: *P* < 0.05 compared with preoperative baseline, generalized estimated equations using an unstructured working correlation matrix structure; Mo: months.

**Figure 3.**
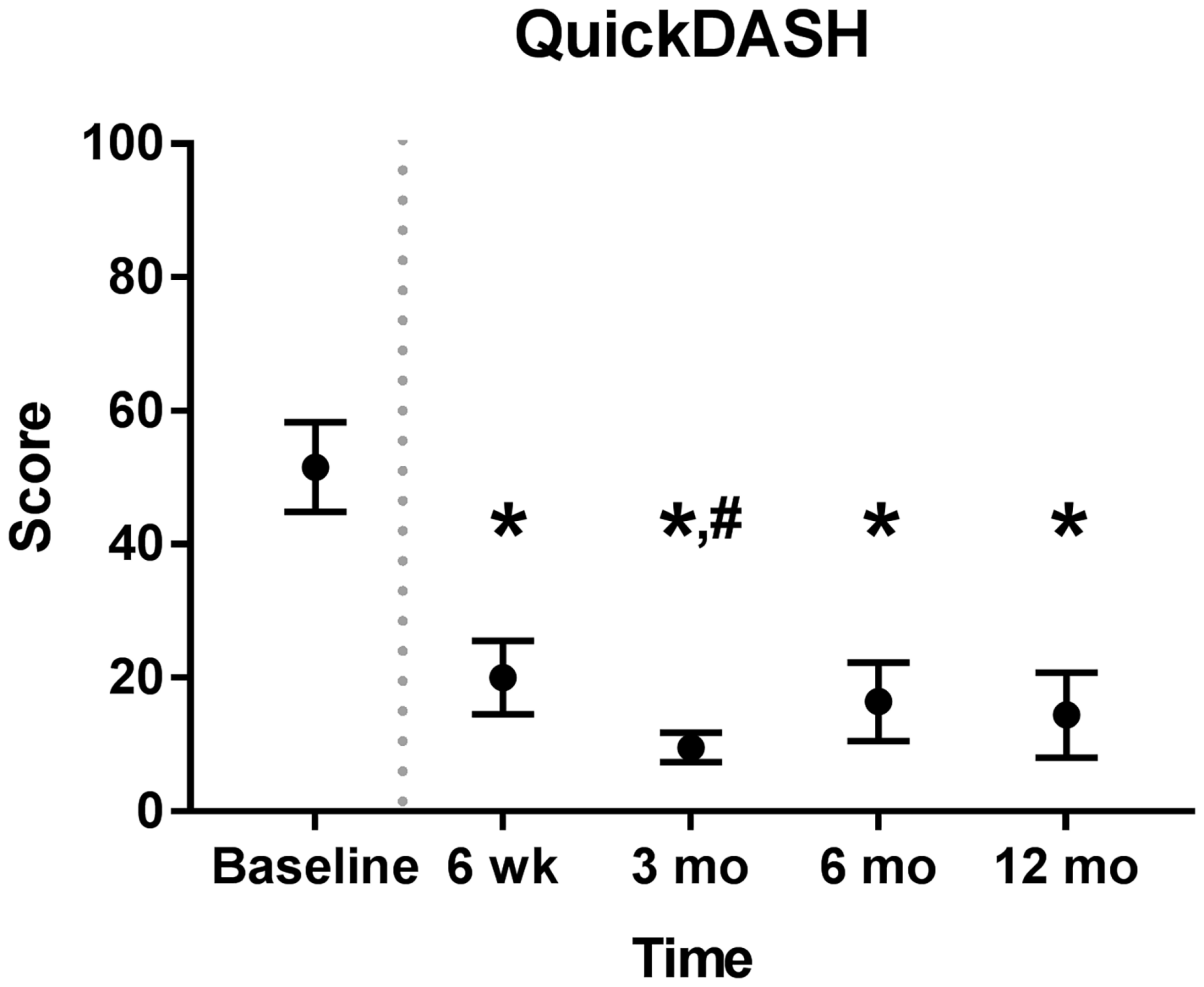
Functional improvement of upper limb after neuroma excision and capping with NEUROCAP®, as measured by Quick Disabilities of the Arm, Shoulder and Hand (QuickDASH) questionnaire. Dotted grey line represents moment of treatment. Measurements at all time points were for 10 patients, except for 6 and 12 months (*n* = 9). Data represented as estimated marginal means with standard deviation. *: *P* < 0.05 compared with preoperative baseline; #: *P* < 0.05 compared with 6 weeks follow-up, generalized estimated equations using an unstructured working correlation matrix structure; Mo: months.

This prospective, multicentre, single-arm trial was registered at ClinicalTrials.gov (NCT02528266). Patients with upper-limb peripheral symptomatic end-neuroma were enrolled between February 2016 and March 2017 at three Dutch hospitals. Inclusion and exclusion criteria are listed at ClinicalTrials.gov. A total of 10 patients met the inclusion criteria. Eight patients had an end-neuroma of the superficial radial nerve (SRN) and two of the median nerve. Preoperatively, the locations of the end-neuromas were confirmed by Tinel's test and a decrease in pain after lidocaine block, as demonstrated by a four-fold reduction in the visual analogue scale (VAS) for pain, from 80 to 20 (*p* < 0.05). Intraoperatively, the neuromas were excised, and the nerve end placed into the NEUROCAP® and secured with non-resorbable sutures. The nerve and cap were then surrounded with and secured onto the surrounding soft tissues. Postoperatively, the VAS pain score reduced from 73.3 at baseline to 17.3 at 6 weeks follow-up. An international neuropathic pain score system (Douleur Neuropathique 4 or DN4) was reduced from 6.4 to 3.3 and neuroma pain score (Elliot et al., 2010) reduced from 12.8 to 3.5. All pain scores remained similarly low throughout the 12 months follow-up (Figure 2). At 12 months, the mean VAS score was 22.1, DN4 score 3.4 and neuroma pain score 4.4. Disability of the affected upper limb improved, demonstrated by a decrease in Quick Disabilities of the Arm, Shoulder, and Hand (QuickDASH) questionnaire score from 51.6 at baseline to 20.0 at 6 weeks and 14.4 at 12 months (Figure 3).

Preoperatively, all patients used at least one type of painkillers. On average, the preoperative intensity of each type of neuropathic pain was moderate to severe and for one patient, the worst pain imaginable. After 6 weeks, only three patients required the continued use of painkillers, with the majority reported no pain or only pain of mild intensity. There were no adverse device-related effects, as assessed by an independent Data Safety Monitoring Board. One patient suffered an external trauma within 6 weeks postoperatively, resulting in a seroma, re-operation and removal of the NEUROCAP®. The patient continued follow-up until 3 months and decided to quit the study afterwards. At study exit, the patient had no neuropathic pain and his improved QuickDASH score remained. There were two neuroma recurrences involving the radial sensory nerve, one after an external trauma and the other with an unclear origin; both of which were treated conservatively. Other adverse events that occurred were anticipated and included postoperative pain and numbness of the operated area, inherent to the healing process and did not require intervention.

Our results compared favourably with those of Regal and Tang (2019) and Yao et al. (2017), when focused on the SRN. The use of NEUROCAP® in our patients demonstrated a similar or better result regarding revision surgery rate and improvement of disability. The results were not superior to the technique of end-to-side repair of SRN neuroma, which resulted in pain-free follow-up of at least 16 months in three patients (Regal and Tang, 2019). However, the results of VAS score were comparable with muscle implantation or SRN neurolysis and interposition of acellular dermal matrix allograft (Yao et al., 2017). The major limitation in our study is the small sample size, and future studies should be further assessed within a larger patient group. Future studies should assess more long-term outcomes and compare different treatments for symptomatic end-neuromas, including treatment with the bioresorbable NEUROCAP®, to determine potential benefits for each treatment option. To this end, a prospective clinical trial with a larger group and 2-year follow-up is currently underway (ClinicalTrials.gov number NCT02993276).
